# Predator‐induced shape plasticity in *Daphnia pulex*

**DOI:** 10.1002/ece3.10913

**Published:** 2024-02-05

**Authors:** Sam Paplauskas, Oscar Morton, Mollie Hunt, Ashleigh Courage, Stephanie Swanney, Stuart R. Dennis, Dörthe Becker, Stuart K. J. R. Auld, Andrew P. Beckerman

**Affiliations:** ^1^ Biological & Environmental Sciences University of Stirling Stirling UK; ^2^ School of Biosciences University of Sheffield Sheffield UK; ^3^ Present address: EAWAG Dübendorf Switzerland

**Keywords:** defence, geometric morphometrics, integration, modularity, phenotypic plasticity, predation

## Abstract

All animals and plants respond to changes in the environment during their life cycle. This flexibility is known as phenotypic plasticity and allows organisms to cope with variable environments. A common source of environmental variation is predation risk, which describes the likelihood of being attacked and killed by a predator. Some species can respond to the level of predation risk by producing morphological defences against predation. A classic example is the production of so‐called ‘neckteeth’ in the water flea, *Daphnia pulex*, which defend against predation from *Chaoborus* midge larvae. Previous studies of this defence have focussed on changes in pedestal size and the number of spikes along a gradient of predation risk. Although these studies have provided a model for continuous phenotypic plasticity, they do not capture the whole‐organism shape response to predation risk. In contrast, studies in fish and amphibians focus on shape as a complex, multi‐faceted trait made up of different variables. In this study, we analyse how multiple aspects of shape change in *D. pulex* along a gradient of predation risk from *Chaoborus flavicans*. These changes are dominated by the neckteeth defence, but there are also changes in the size and shape of the head and the body. We detected change in specific modules of the body plan and a level of integration among modules. These results are indicative of a complex, multi‐faceted response to predation and provide insight into how predation risk drives variation in shape and size at the level of the whole organism.

## Graphical abstract

Recent advances in statistical methods for measuring shape were combined with a classic example of continuous phenotypic plasticity to measure the whole‐organism shape response to predation in *Daphnia pulex*. We found complex, multi‐faceted shape changes, with a mixture of locally and globally co‐ordinated features.

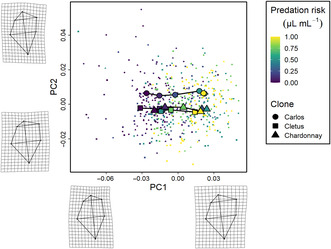

## 1 INTRODUCTION

A major challenge facing all organisms is to adapt to environments that vary within their lifespan. A route to responding to and surviving such variation is phenotypic plasticity, the ability of individual clones to change phenotype during their life cycle when exposed to different environments (West‐Eberhard, 2003). One pervasive, natural source of environmental variation is the risk of being attacked and killed by a predator, known as predation risk.

Predation risk induces a suite of changes in the behaviour, life history and morphology of many plants and animals (Bell & Sih, 2007; Bradshaw, 1965; Dicke & Hilker, 2003; Lürig et al., 2019; Ower & Juliano, 2019; Relyea, 2001) and such predator‐induced responses are considered classic examples of phenotypic plasticity. Of particular interest are morphological responses to predation risk, which range from the production of spines (Arnqvist & Johansson, 1998; Tollrian, 1994) to changes in the shape of a portion of the body (Buskirk, 1998) or the entire body plan (Brönmark & Miner, 1992) of an organism.

Many studies of predator‐induced change have focussed on a linear assessment of shape, measuring the distance between two points. Classic examples include changes in defensive dorsal spine length (Januszkiewicz & Robinson, 2007), body depth that affects vulnerability to gape‐limited predators (Brönmark & Miner, 1992) and morphological features associated with behavioural swimming escape responses (Domenici et al., 2008). However, this type of linear analysis only captures a subset of the overall shape variation.

A more complete understanding of the response to predation requires an assessment of overall shape, which requires a multivariate approach (the measurement of multiple different landmarks defining shape, at once). A well‐established method for assessing multivariate plasticity in shape is geometric morphometrics (Rohlf, 1998), which uses anatomical coordinates as shape variables to measure relative differences in shape.

While this approach has been used to measure predator‐induced changes in shape for a wide range of organisms, such as fish (Arnett & Kinnison, 2016; Díaz‐Gil et al., 2020; Franssen, 2011), amphibians (Florencio et al., 2020; Reuben & Touchon, 2021; Ruehl et al., 2018) and snails (Hooks & Padilla, 2021; Solas et al., 2015; Terry & Duda, 2021), until recently, shape has rarely been assessed as a plastic trait in water fleas (*Daphnia* species), an iconic organism for the study of size‐selective, predator‐induced phenotypic change. Instead, daphnid research has largely focused on scoring the production of inducible morphological defences, such as the head spikes of *Daphnia pulex*, called ‘neckteeth’, which develop in response to predator cues (kairomones) released from their midge larvae predators (Krueger & Dodson, 1981; Parejko & Dodson, 1991; Tollrian, 1993).

These studies have highlighted the importance of the neckteeth defence as a model for continuous phenotypic plasticity. They have shown that the strength and duration of induction varies according to the stage of development and depends on the concentration of predator cue (Beckerman et al., 2010; Naraki et al., 2013; Weiss et al., 2016). Evidence also suggests that there is a threshold for adaptive phenotypes to evolve, which is driven by the trade‐off between the fitness costs and benefits of neckteeth production under different levels of predation risk (Hammill et al., 2008). Importantly, several studies also indicate that changes in morphology form part of an integrated phenotypic response to predation risk (sensu Plaistow & Collin, 2014), which also includes changes in size and age at maturity (Beckerman et al., 2010). Given this evidence of sensitivity to the environment, the fitness costs/benefits and integration with a variety of traits, it is somewhat surprising how inconclusive our understanding of the overall shape response of *D. pulex* to predation risk is.

In particular, unresolved questions centre on the modularity and integration of the body plan under predation risk. For example, are there modules in the body plan and what is the level of coordination in the development of morphological structures (modules; Klingenberg, 2014)? Answering these types of questions using geometric morphometrics helps to identify functional relationships between traits (Klingenberg et al., 2010; Martinez & Sparks, 2017; Zelditch & Goswami, 2021), constraints on phenotypic responses (Du et al., 2019; Klingenberg, 2005; Sanger et al., 2012) and the cost and benefits of alternative phenotypes (Martín‐Serra et al., 2019, 2021; Pedraza‐Pohlenz et al., 2023).

In this study, we evaluate shape plasticity along a gradient of six levels of predation risk in three clones of *D. pulex* which differ in their sensitivity to predator cues. In this intraspecific, fine gradient analysis, we apply morphometric landmark‐based methods to photographs taken by Dennis et al. (2011), in which *D. pulex* were exposed to six levels of predation risk (and a control) from their midge larvae predator, *Chaoborus flavicans*. We combine geometric morphometrics with phenotypic trajectory analysis to formally evaluate the multivariate change in shape and estimate measures of both modularity and integration to evaluate if there are coherent units of the body plan that respond to predation risk, and whether the development of these units is independent or highly co‐ordinated (Adams & Collyer, 2009; Collyer & Adams, 2007; Dennis et al., 2011).

There are two recent studies of predator‐induced shape in *D. pulex* that invoke module identification using morphometrics and parallel the work we present here. A recent study by Becker et al. (2022) showed in *D. pulex* some evidence to suggest the presence of modularity in the development of the neckteeth defence along the dorsal region of the carapace. Here, the researchers combined morphometrics of the dorsal region of *D. pulex* under control and predation risk with molecular genetic analysis to distinguish among stabilising or diversifying selection driving the induced defence (Becker et al., 2022). This work identified dorsal modules that possessed more or less genetic variation as part of this analysis. In another recent study, Horstmann et al. (2021) introduced three‐dimensional laser scanning to deliver a comparison of overall shape response to predation risk among multiple species of *Daphnia* and multiple predators. This work demonstrates prey–predator interspecific changes in body depth, head thickness and various other changes which could potentially be linked to avoiding gape‐limited predation, enhanced swimming or increased body stiffness (Horstmann et al., 2021).

As noted above, and in contrast to the above studies, we present here an intraspecific, fine scaled analysis of modularity and integration of the body plan of *D. pulex* under a gradient of predation risk. In advance of the analysis, we have three main hypotheses. Our first hypothesis is that wider bodies and bigger heads will form part of the predator‐induced response in *D. pulex*. This is because wider bodies may help release *D. pulex* from gape‐limited predation by *Chaoborus* (Pastorok, 1981) and bigger heads are more likely to interfere with predation by increasing the effectiveness of the neckteeth defence. Our second hypothesis is that there will be modularity in the head and body regions, where they are separated in their response to predation risk, with little to no integration between them, due to the functional differences in how each part of the animal defends against predation. Our third hypothesis is that there will be no modularity in the ventral and dorsal regions, but there will be integration between them, due to the expected fitness benefits of co‐ordinating the response of multiple traits with similar functions to spatial and/or temporal variation in the environment.

## 2 MATERIALS AND METHODS

To evaluate our hypothesis about the nature of the predator‐induced shape change in *D. pulex*, we first analysed the phenotypic trajectories (Collyer & Adams, 2013) of three *D. pulex* clones along a gradient of predation risk, focusing on the magnitude, direction and shape of the change in multivariate trait space. Second, to evaluate our hypotheses about the relationship between different aspects of predator‐induced shape change, we performed modularity and integration tests on the head‐body and dorsal‐ventral parts of the animal. In the following sections, we introduce the study system, data and design before providing further details on the analyses mentioned above.

### 2.1 Study system

**FIGURE 1:**
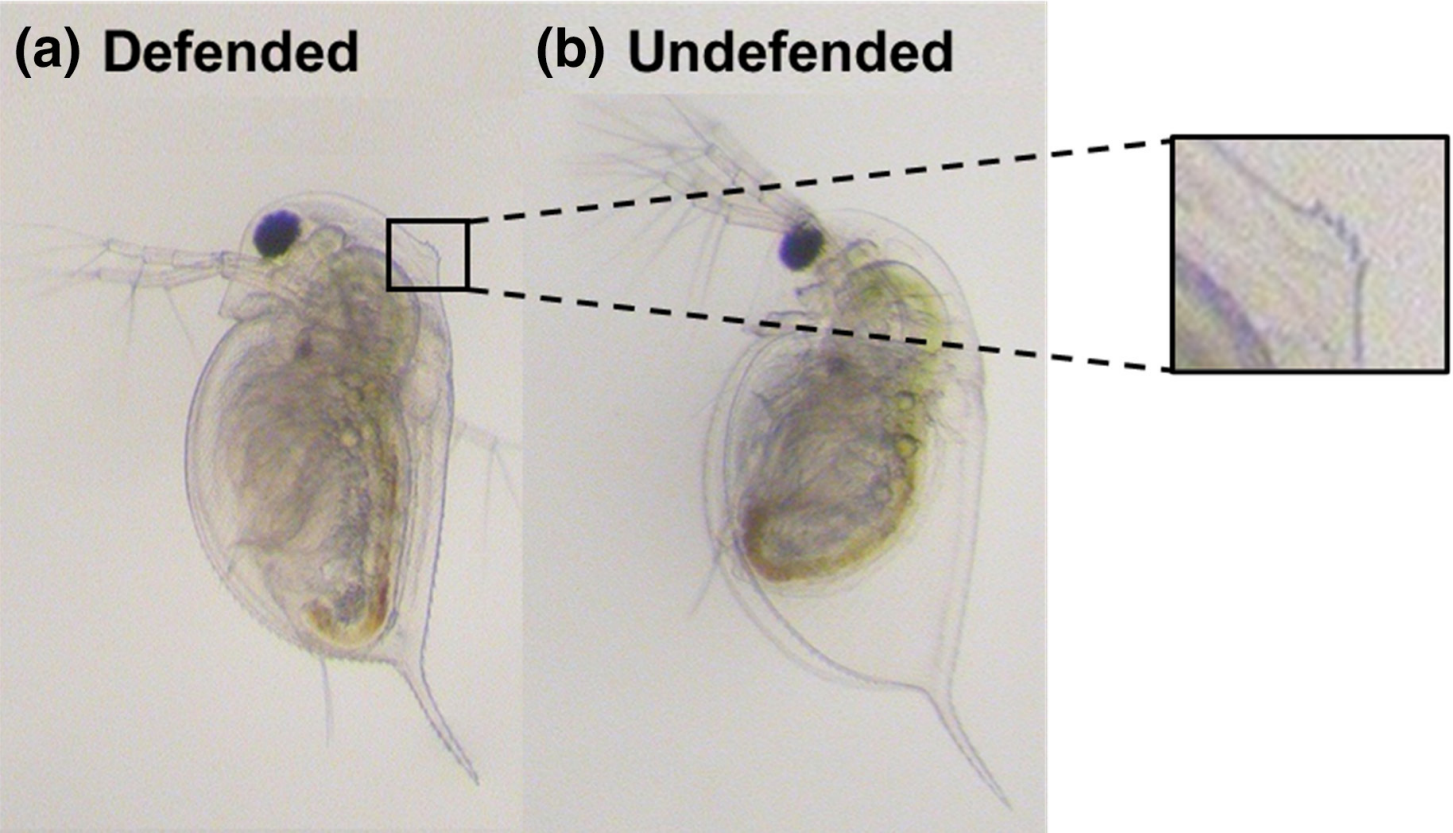
(a) Defended and (b) undefended morphs of *Daphnia pulex*. Animals are third instar juveniles exposed to either 1 or 0 μL mL^−1^ (control) of *Chaoborus flavicans* predator cue. The enlarged image shows a close‐up of the neckteeth defence.

In this study, we focus on one of the most iconic predator‐induced defences in water fleas (*Daphnia* species), the ‘neckteeth’ of *D. pulex* (Figure 1). The neckteeth defence is composed of a swollen area on the back of the head (neck‐pedestal) and spikey projections which grow on top. The defence grows in response to predator cues (kairomones) released by midge larvae predators (*Chaoborus* spp., Parejko & Dodson, 1991; Tollrian, 1993), starting with the development of the neck‐pedestal, which first begins to grow during the late embryonic stage at the onset of kairomone sensitivity, followed by the defensive head spikes which develop later in the early juvenile stages (Naraki et al., 2013; Weiss et al., 2016). The maintenance of the defence requires consistent exposure to predator cues (Imai et al., 2009) and usually lasts until the third instar, after which the *Daphnia* are large enough to escape size‐selective predation (Tollrian, 1993). Also, it is well known that the induction of the defence increases with the level of predation risk (Carter et al., 2017). Although the exact mechanism is unclear, it has been shown that the neckteeth defence reduces the total number of predator strikes and increases the likelihood of escape (Kruppert et al., 2019), and as a result, increases prey survival by up to 50% (Hammill et al., 2008).

### 2.2 Study design

We used data originally collected by Dennis et al. (2011). This included photographs of second and third instar *D. pulex* that had been exposed to six different concentrations of *C. flavicans* kairomone, including a control (0, 0.1, 0.25, 0.5, 0.75 and 1 μL mL^−1^), which was extracted from frozen larvae (Honka, Germany), following the method developed by Tollrian (1995). For each treatment, third‐generation mothers of at least their third brood were exposed to the relevant cue concentration and then five offspring from the three to four subsequent broods were transferred to glass jars which contained 50 mL of hard artificial pond water (American Society for Testing Materials, 2007), food (2 × 10^5^ cells mL^−1^ of *Chlorella vulgaris*) and the appropriate concentration of predator cue. These animals were transferred to a new jar, containing fresh media and cue, and lateral‐view photographs were taken every day with a Canon EOS DLSR mounted to Leica MZ‐9 stereomicroscope. Live specimens were placed under the microscope in approximately the same position to minimise error due to parallax (Mullin & Taylor, 2002), and all images were acquired with the same magnification. However, only photographs taken at the second and third instar were used in this study, because this is when the defence is usually at the peak of induction (Tollrian, 1993).

From this data set, we analysed three clones for shape plasticity. According to the reaction norms of the predator‐induced phenotype shown in Dennis et al. (2011), we selected two clones (Chardonnay and Cletus) that showed a characteristically low level of neckteeth induction and one (Carlos) a characteristically high level. We also pooled the photographs of animals taken from across different broods to form a factorial design of three clones × two instars × six predation risk levels. There were 4–20 replicate photos (i.e. replicate individuals) at each level of predation risk for each clone (totalling 518 photos).

### 2.3 Digitisation

**FIGURE 2:**
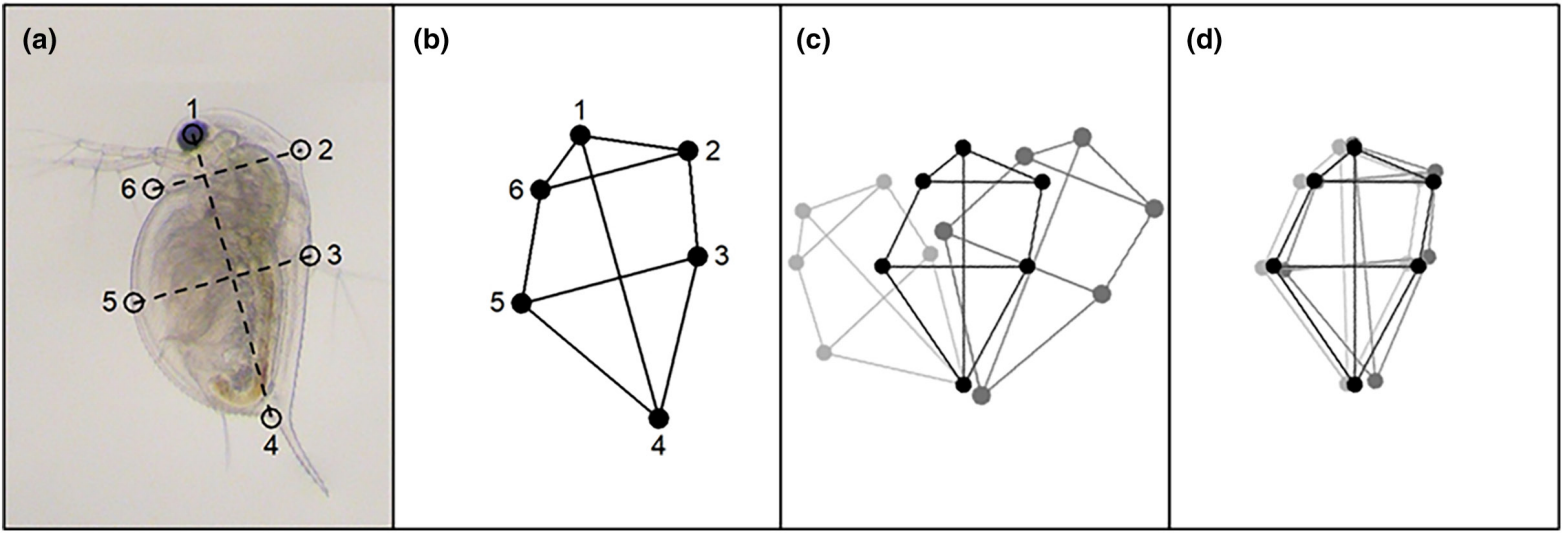
Image digitisation and Procrustes superimposition. (a) First, images were prepared for landmarking in Microsoft Paint. This process identified a total of six landmarks for each photo (open circles) including (1) the centre of the eye, (2) the neckteeth defence (or the corresponding area in the controls), (3) the dorsal midpoint of the carapace, (4) the base of the apical spine, (5) the ventral midpoint of the carapace and (6) the rostrum. (b) Second, the position of the landmarks (closed circles) was recorded manually in R to create a digitised image of each specimen, represented by a set of *X* and *Y* coordinates, that captured key aspects of shape (links between circles). (c) Third, since digitised images varied in in terms of size, position and orientation, which is shown by the differences between each aggregation of coloured circles (painted black or lightly transparent / dark grey), they were aligned using Generalized Procrustes Analysis from the *geomorph* package v4.0.5 so that differences in shape could be compared. (d) The final set of digitised images.

We digitised images using the geometric morphometric method (Rohlf, 1998) to create a few select two‐dimensional anatomical co‐ordinates (reference points, called ‘landmarks’) that characterised body shape. First, lateral‐view photographs were uniformly orientated (head at the top) and mirrored (so that the animals were facing in the same direction) to prepare for landmarking in Microsoft Paint. This involved drawing a vertical line from the eye to the base of the apical spine (a standard measure of daphnid length) and then two perpendicular lines, one at the midpoint of the original line and another intersecting the rostrum. These lines provided a method to consistently capture the approximate location of the dorsal and ventral midpoints between different images, as well as the region on the back of the neck where defences were induced (Figure 2a). This process identified a total of three fixed landmarks and three semi‐landmarks for each photo, including (1) the centre of the eye, (2) the neckteeth defence (or the corresponding area in the controls), (3) the dorsal midpoint of the carapace, (4) the base of the apical spine, (5) the ventral midpoint of the carapace and (6) the rostrum. The choice of these landmarks was motivated by a method developed for linear dimensions in two species of *Daphnia*, *D. dentifera* and *D. mendotae* (Duffy et al., 2004) and were selected to capture key aspects of the defence, head and body shape.

Images were then digitised by applying landmarks using the *geomorph* package v4.0.5 in R v4.2.1 (Figure 2b, Adams et al., 2022; Baken et al., 2021). Before these landmarks were analysed in R, they were standardised using generalised Procrustes superimposition from the *geomorph* package v4.0.5 (Figure 2c, Adams et al., 2022; Baken et al., 2021). This process repositioned the landmarks to rectify inconsistency among replicates due to uncontrollable differences in the size, position and orientation of the original specimen so that shape could be compared in a meaningful way. The resulting superimposed Procrustes shape coordinates (Figure 2d) were used in the subsequent analyses.

The repeatability of selecting landmarks was measured using all 518 photographs. Each photograph was measured once in two separate digitising sessions and the mean squared (MS) error was calculated from Procrustes‐aligned shape coordinates. The repeatability was calculated from the difference between the MS relating to the individual and the MS relating to the digitising session and then calculating the ratio of this value to the total MS (Zelditch et al., 2004).

### 2.4 Data analysis

To test the statistical significance of variation in shape across different factors, including size (the sum of the squared distances of each landmark to the mean position of all the landmarks for an individual specimen), instar, clone and predation risk, we performed Procrustes ANOVA using the procD.lm() function from the geomorph package v4.0.5 (Adams et al., 2022; Baken et al., 2021). The Procrustes ANOVA used a permutation procedure of 10,000 iterations to assess the importance of variation in shape across the different factors for our set of Procrustes‐aligned coordinates.

To further understand the relationship between different factors, specifically clone and predation risk, we performed trajectory analysis using the trajectory.analysis() function in RRPP package v1.3.1 for R (Collyer & Adams, 2018, 2021). The phenotypic trajectory analysis measured morphological variation between treatments in terms of its magnitude (distance moved in shape space), direction (angle of the change in shape space) and shape (relative position of the change in shape space). The mean phenotypic trajectories were visualised using principal component analysis and were connected in the order of increasing predation risk. Thin‐plate spline deformation grids were used to describe the principal component axes by indicating the difference between the mean location of each landmark in the data set and the minimum and maximum locations of each landmark in the data set (see Adams & Collyer, 2007, 2009; Collyer & Adams, 2007, 2013; Dennis et al., 2011).

We evaluated modularity and integration of morphological (co)variation using the covariance ratio (CR, Adams, 2016) and partial least‐squares (PLS) analysis (Bookstein et al., 2003). The CR is a ratio of the overall covariation between modules relative to the overall covariation within modules. The significance of the CR is tested by comparing the value from the actual data to a distribution of CR values obtained by randomly assigning landmarks into subsets across modules. A significant result, which indicates modularity, is found when the observed CR is small relative to this distribution.

When used with landmark data, PLS analysis is referred to as singular warps analysis (Bookstein et al., 2003). The analysis calculates normalised composite scores (linear combinations), one from the X‐variables and one from the Y‐variables, that have the greatest mutual linear predictive power. Similar to the test for modularity, the observed PLS value is compared to a distribution of values obtained by randomly permuting the individuals (rows) in one set relative to those in the other. A significant result, which indicates integration, is found when the observed PLS correlation is large relative to this distribution.

We applied the CR and singular warps analyses across clones and predation risk levels with 999 iterations to test for two non‐mutually exclusive patterns of modularity and integration between (1) the head (rostrum, eye, neck) and lower body (mid‐ventral, spine, mid‐dorsal) regions, and (2) the dorsal (eye, neck, mid‐dorsal) and ventral (rostrum, mid‐ventral, spine) regions. We also visualised the changes in the different sets of landmarks using thin‐plate spline deformation grids (see earlier references).

### 2.5 Ethics statement

We confirm that the methods employed in this study were reviewed and approved by the institutional review committee and all animals from the original experiment were cared for in accordance with institutional and national guidelines.

## 3 RESULTS

### 3.1 Repeatability

First, there was absolutely no error introduced by observer bias because all the photos were digitised by the same individual. Second, the repeatability of measuring shape by applying landmarks to photos of individual specimens was very high, 93.1% (mean squared error).

### 3.2 Predation risk alters shape

Analysis of *Daphnia* shape using Procrustes ANOVA revealed that shape varied across all factors, including size, instar, clone and predation risk (all *p* < .001). It also showed that the response to the level of predation risk did not vary by instar (predation × instar interaction; *F* = 1.39, *df* = 5, *p* = .13), but it did vary by clone (predation × clone interaction; *F* = 4.92, *df* = 10, *p* < .001).

**FIGURE 3:**
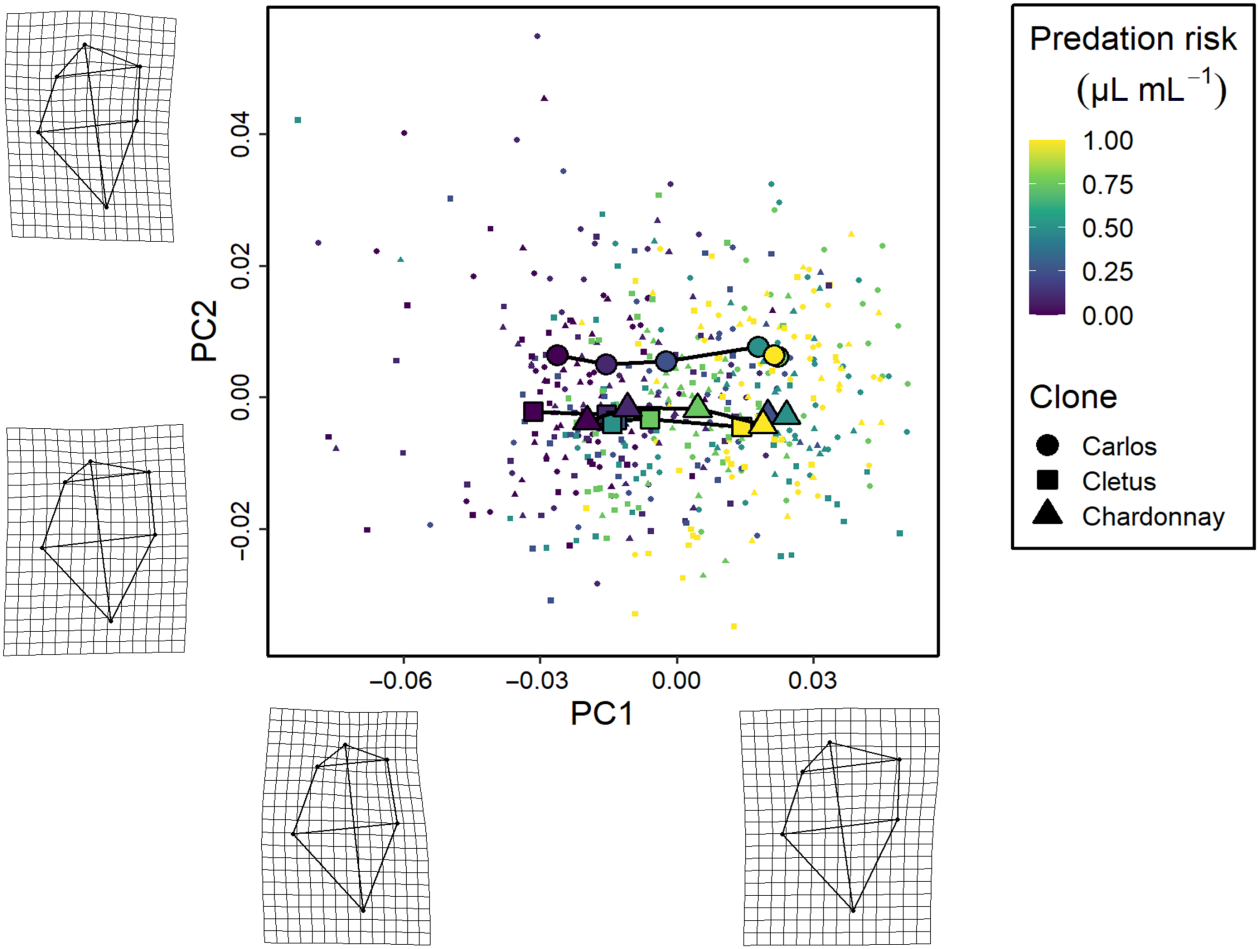
The first set of three *Daphnia pulex* phenotypic trajectories along a gradient of predation risk. The principal components summarise changes in neck shape (PC1) and head height (PC2), which are visualised by the deformation grids along the axes. Individual data points for each specimen are shown by the small points, whereas the large points correspond to the mean phenotype for each treatment. The colour indicates the level of risk (0, 0.1, 0.25, 0.5, 0.75 and 1 μL mL^−1^) and the lines connect each treatment in order of increasing predation risk. The shape of the points refers to the specific clone used in the experiment (circles—Carlos, squares—Cletus, triangles—Chardonnay).

**FIGURE 4:**
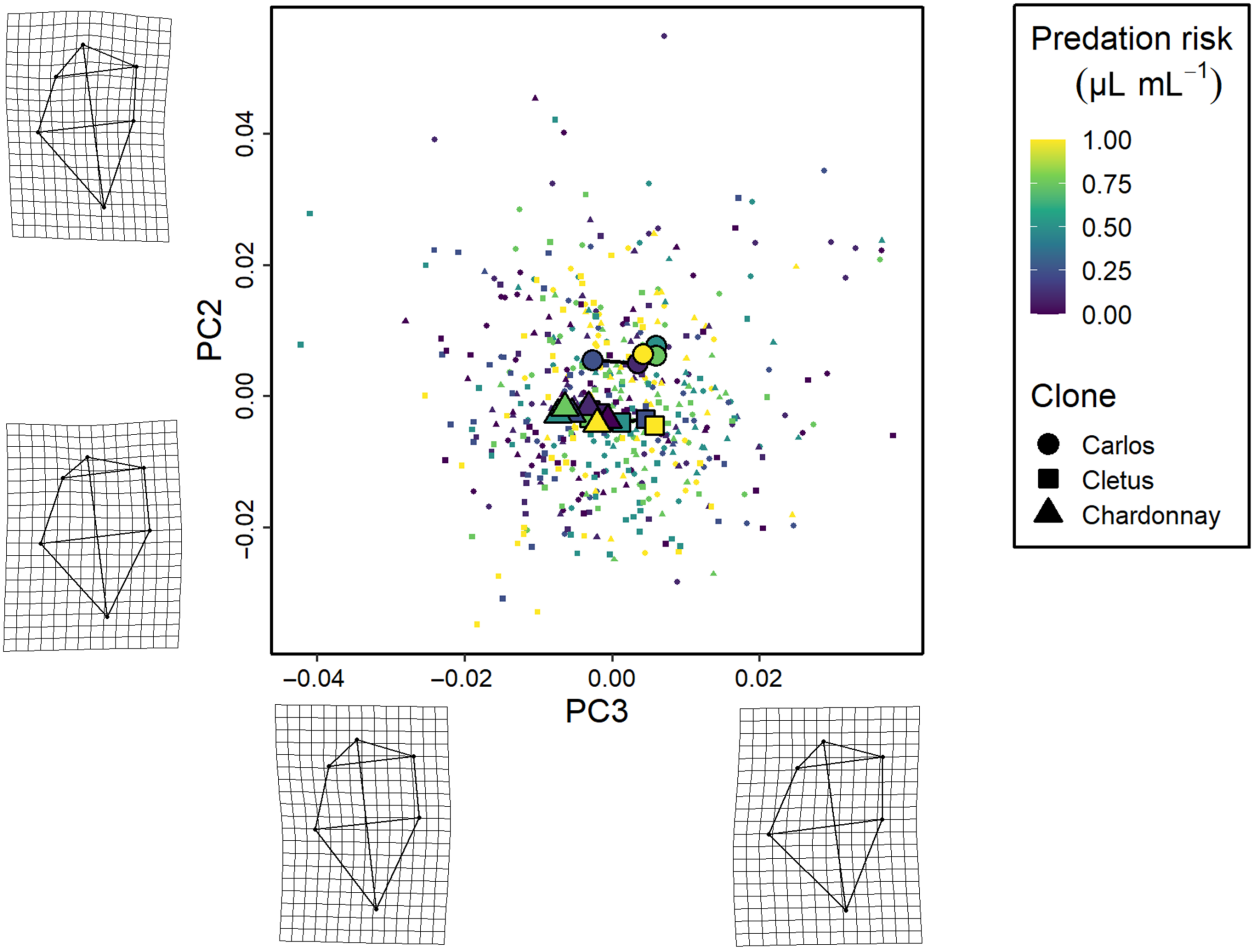
The second set of three *Daphnia pulex* phenotypic trajectories along a gradient of predation risk. The principal components summarise changes in carapace bulge (PC3) and head height (PC2). See the description of Figure 3 for the legend.

**FIGURE 5:**
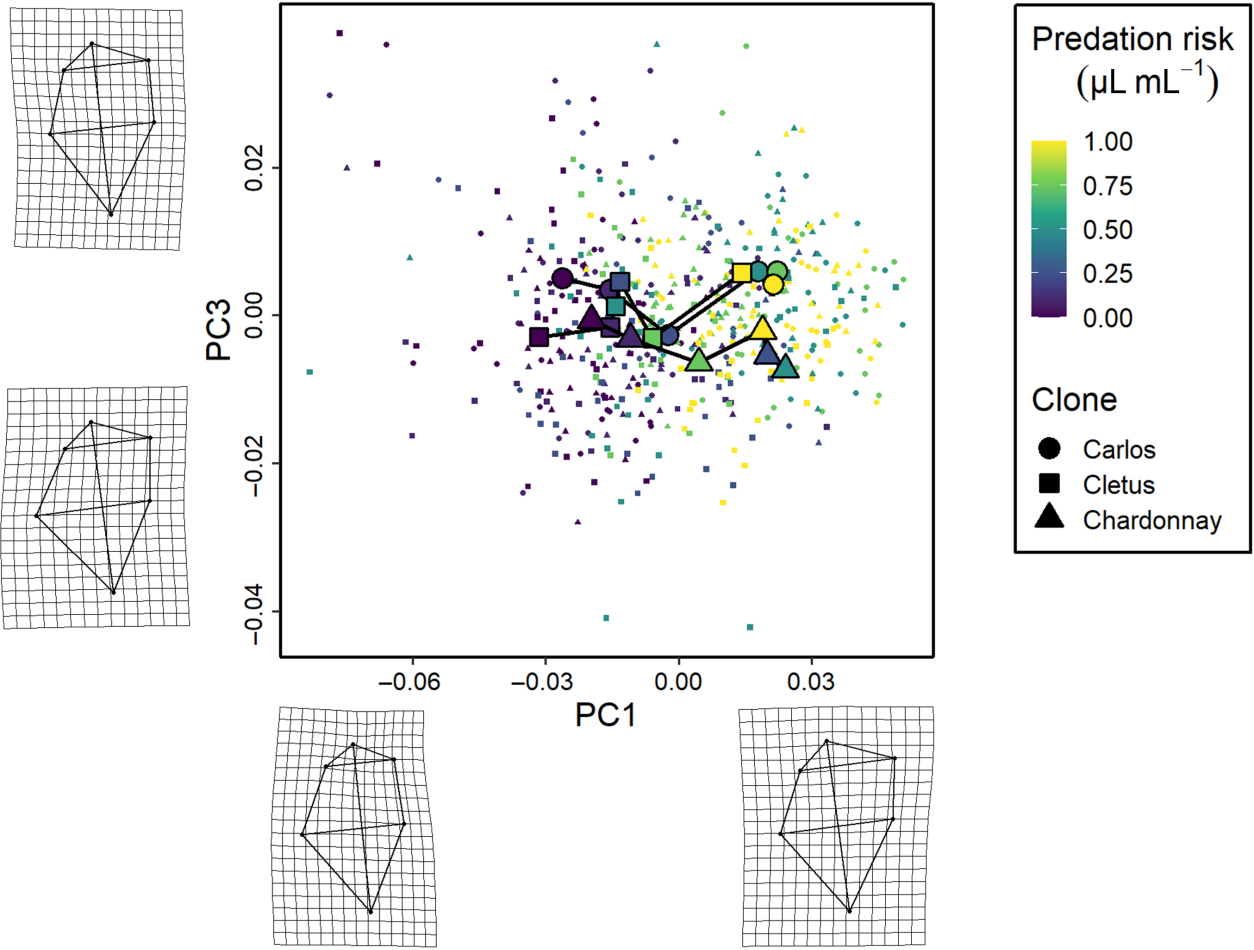
The third set of three *Daphnia pulex* phenotypic trajectories along a gradient of predation risk. The principal components summarise changes in neck shape (PC1) and carapace bulge (PC3). See the description of Figure 3 for the legend.

The phenotypic trajectory analysis showed that the interaction between the level of predation risk and clone was not dependent on how much the clones responded to predation cues (path distances were equivalent, all pairwise differences *p* > .05), but around differences in the direction of these changes in multivariate trait space (the angle of the change in shape space) and the shape of these changes (the relative position of the changes in shape space, all pairwise differences *p* < .01) along the six cue concentration gradient (Figures 3, 4, 5). Specifically, the direction of change in clone ‘Cletus’ differed from both clone ‘Chardonnay’ and ‘Carlos’ (both *p* < .05) indicating that the landmarks that changed in ‘Cletus’ were different than in the other two clones, which is reflected in the slightly different path that the trajectory of each clone takes along the cue concentration gradient (Figure 5).

#### 3.2.1 Neckteeth, head height and carapace bulge

The principal component analysis indicated that 55% of the variation in shape was captured by PC1, 16% by PC2 and 12% by PC3 so the first three PCs described 83% of the overall shape variation in total. A visualisation of the trajectories in 2D PC space, along with details on what kind of shapes were associated with the PC‐axes, revealed several key insights.

First, based on the deformation grids associated with the PC axes, we were able to define PC1 as neck‐change (i.e. the inducible defence), PC2 as head height and PC3 as ventral carapace bulge (Figures 3, 4, 5). Second, the trajectories of all three clones moved in parallel to PC‐axis 1, which showed that the neck region was larger when there was more cue (Figures 3 and 5). Third, there were clear differences among the clones linked to head height (PC2 axis).

### 3.3 Head and body shape is modular, but there is integration across all body regions

We assessed the developmental link between two sets of landmarks using the modularity and integration tests. First, we assessed the link between the head (rostrum—eye—neck) and body regions (mid‐ventral—spine—mid‐dorsal). Second, we assessed the link between the dorsal (eye—neck—mid‐dorsal) and ventral (rostrum—mid‐ventral—spine) regions.

**FIGURE 6:**
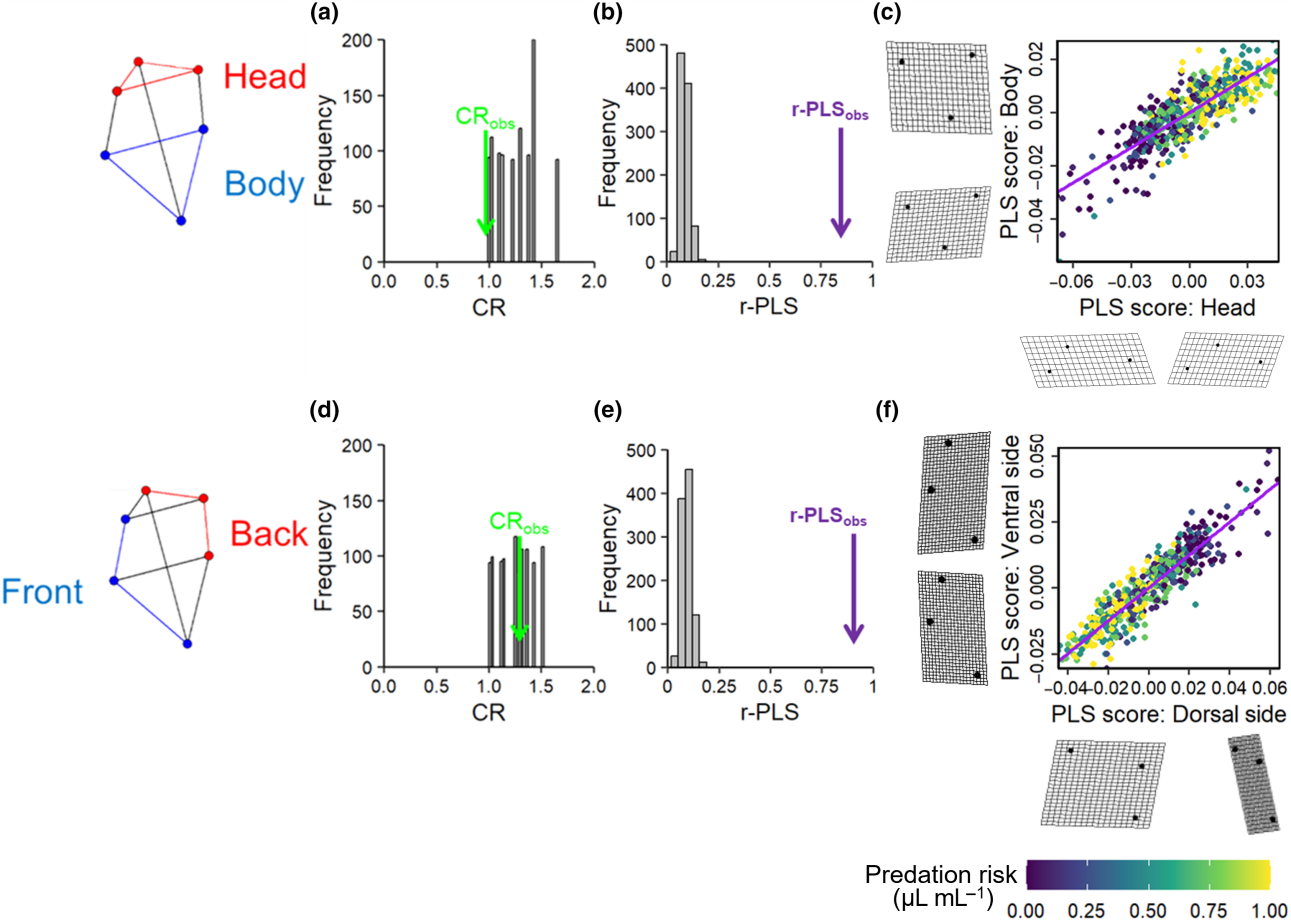
Modularity and integration tests. (a) Histogram of covariance ratio (CR) coefficients produced from simulations of random partitions of the head and body, with the observed CR coefficient indicated by the green arrow. (b) Histogram of partial least squares analysis correlation coefficients (r‐PLS) produced from simulations of random covariation between the head and body, with the observed r‐PLS indicated by the purple arrow. (c) Observed PLS scores for the head and body, with deformation grids that visualise the shape change along the axes and the line of best fit (purple line). (d–f) The same as (a–c), but for the ventral and dorsal regions. The specific groups of landmarks involved in the tests are represented by the diagrams at the left of the plots. The presence of modularity is indicated by an observed CR coefficient that is small relative to the distribution of randomly permuted values, whereas integration is indicated by an observed PLS correlation that is large relative to the distribution of randomly permuted values. The colour of the points indicates the level of predation risk for the observed PLS scores, including 0, 0.1, 0.25, 0.5, 0.75 and 1 μL mL^−1^.

The first set of tests showed evidence of modularity in the head and body regions by indicating not only a high level of covariation between traits within modules (CR = 1, CI = 0.00, *p* < .05; Figure 6a) but also a significant level of integration (covariation) between them (r‐PLS: 0.825, *p* = .001; Figure 6b). This suggests that there is a high level of coordination between the shape variables within the head and body regions, and they also change in an integrated manner. Specifically, as the neck region enlarged, the dorsal midpoint of the carapace moved downwards in a caudal direction, the spine was drawn towards the posterior and the rostrum shifted inwards in a proximal direction (Figure 6c).

In comparison, the second set of tests showed no evidence of modularity in the ventral and dorsal regions (CR = 1.29, CI = 0.00, *p* = .54; Figure 6d), but there was evidence of integration between them (r‐PLS: 0.917; *p* = .001; Figure 6e). This suggests that these two groups of landmarks respond to predation risk in a highly co‐ordinated manner. These integrated changes were very similar to those described above; as the neck region was expanded by the inducible defence, the dorsal midpoint of the carapace moved downwards in a caudal direction, the spine was pulled towards the posterior and the rostrum was pulled inwards in a proximal direction (Figure 6f).

## 4 DISCUSSION

Our understanding of how organisms respond to predation risk has traditionally focused on a small number of specific traits in only a few environments (Day & Rowe, 2002; Heino et al., 2002; Kishida et al., 2010; Roff, 1999). Recent advances have moved the standard of plasticity research to a multi‐trait approach including morphology, life history and behaviour along environmental gradients (Barbasch & Buston, 2018; Bourdeau, 2012; Chiaverano et al., 2016; Dennis et al., 2011; Dijk et al., 2016; Forsman, 2015; Heynen et al., 2016; Reger et al., 2018). This has been complemented by a growing appreciation that morphometric analyses applied to organism shape can provide added value to analyses of phenotypic plasticity.

Here, building on this growing use of morphometrics in plasticity research, we evaluated ‘shape’ plasticity among three clones of *D. pulex* exposed to a gradient of six levels of predation risk. Our objective was to use morphometric shape as a ‘summary’ trait affected by responses to predation risk in life history and morphology to evaluate several hypotheses about the impact of predation risk on whole organism plasticity. Our motivation was linked to size selective predation theory (Abrams & Rowe, 1996; Dmitriew, 2011; Preisser & Orrock, 2012; Taylor & Gabriel, 1992; Tollrian & Harvell, 1999) and work on the response of fish to predation risk where size and shape are both linked to survival (Arnett & Kinnison, 2016; Brönmark & Miner, 1992; Díaz‐Gil et al., 2020; Domenici et al., 2008; Franssen, 2011; Januszkiewicz & Robinson, 2007). We found that the *D. pulex* response to predation risk involves both modular and integrated changes, with changes in the inducible neckteeth defence linked to changes in head and body shape. Although we focussed on only a small number of shape variables, this suggests that there is a complex response to predation, including strong developmental correlations in how *Daphnia* body plans are organised.

Our first set of results from the trajectory analysis showed how morphology changed in response to a gradient of predation risk. In accordance with previous studies of the inducible defence (Dennis et al., 2011; Laforsch & Tollrian, 2004; Tollrian, 1993) and our initial hypothesis, we found that head width (neck‐change) increases along the gradient of predation risk. We also found that head height varied among clones and body width (carapace bulge) was a non‐linear feature of change. This last result is contrary to our initial hypothesis; we predicted that body width would increase along a rising gradient of predation risk due to gape‐limited predation, but in reality, body width varies across the different levels of predation risk used in this study.

Although the importance of the neckteeth defence for *D. pulex* survival against predation by *Chaoborus* midge larvae has been shown before (Dennis et al., 2011; Laforsch & Tollrian, 2004; Tollrian, 1993), there has been relatively little research on changes in head height and body width. In terms of variation in head shape, one previous study found a decrease in lateral head width in defended compared to undefended morphs of *D. pulex* (Horstmann et al., 2021) and it was suggested that, together with an increased thickness around the heart region, this may result in increased stiffness that protects against deformation during predation. We suggest that the variation in head shape, something which was also observed in our study, could be linked to hydrodynamic drag and the streamlining properties of *D. pulex* during swimming and the evasion of predators. This has been shown to be the case for other aspects anti‐predator defensive morphology in the closely related *Bosmina* species (Lagergren et al., 2000; Lord et al., 2006).

Regarding changes in body shape, two previous studies using linear morphometrics showed a relatively small increase in body width in defended compared to undefended morphs of *D. pulex* (Tollrian, 1995) and *D. magna* (Rabus & Laforsch, 2011), which suggests that body width may play a minor role in the response to predation. In support of this, it has been shown that body width is a better predictor for prey size range in *Chaoborus* than body length (Swift, 1992). This is because *Chaoborus* usually swallows prey that cannot be deformed only if its diameter is not wider than the larva's head capsule diameter (Swift, 1992). Alternatively, the increase in body width may result from the increase in strength, and possibly thickness, of the carapace observed in defended morphs of *D. pulex* and other *Daphnia* species (Kruppert et al., 2017; Laforsch et al., 2004).

Our second set of results from the modularity and integration tests showed evidence for developmental modularity in the head and the body (which was as predicted), no modularity in the ventral and dorsal regions (predicted), but a significant level of integration across both sets of shape regions. These results suggest that there is a high level of coordination within the head and the body, and that there is also an integrated ‘trade‐off’ among aspects of the body plan under predation risk. Specifically, as the neck region enlarges, the dorsal midpoint of the carapace moves downwards in a caudal direction, the spine is drawn towards the posterior and the rostrum shifts inwards in a proximal direction.

This combination of modular and integrated changes is indicative of a whole‐organism, integrated response to predation (sensu Forsman, 2015). This is entirely possible, as modularity and integration are not co‐independent. Both modularity and integration can co‐occur by finding modules (from the rejection of a null model of no covariation within modules) and integration between these modules (from the rejection of a null hypothesis of no covariation between modules). The evolution of modularity and integration is often linked to trait functionality. In the case of modularity, there can be selection for ‘variational adaptation’, where traits that often respond together to environmental pressures, such as predation risk, are integrated into one module, and traits that rarely need to be changed at the same time are packed into another module (Wagner et al., 2007). This may explain why changes in the head of *D. pulex*, which form the main response to predation risk, are relatively independent of changes in the body. In terms of the observed integration, one possible function could be to increase fitness by co‐ordinating the response of multiple traits to spatial and/or temporal variation in the environment. Although it is not entirely clear how, this could be linked to greater carapace stability (Laforsch et al., 2004) or an improved escape response (Dodson et al., 1995), but further investigation will be required to understand the precise costs/benefits of these integrated shape changes and the nature of these developmental constraints (correlations).

In this study, we investigated the relationship between the development of different aspects of predator‐induced shape in *D. pulex*. We exposed animals to six levels of predation risk and evaluated shape plasticity using geometric morphometrics and phenotypic trajectory analysis. We now have a better understanding of the multivariate response of *D. pulex* to predation risk by showing that there is genetic variation in this response and that changes can be both modular and integrated, with associated adaptive and non‐adaptive (constraint) hypotheses in need of further evaluation. Thus, there are two clear ‘next‐steps’. The first is to establish the adaptive costs/benefits of shape change and variation. The second is the molecular ecology of the developmental constraints. The availability of genomic tools, the clonal nature of *Daphnia*, well‐established experimental protocols and recent high throughput image analysis (Becker et al., 2022) are an outstanding platform for future research.

## AUTHOR CONTRIBUTIONS

**Sam Paplauskas:** Conceptualization (supporting); data curation (lead); formal analysis (equal); funding acquisition (lead); investigation (equal); validation (lead); visualization (lead); writing – original draft (lead); writing – review and editing (equal). **Oscar Morton:** Conceptualization (supporting); investigation (equal). **Mollie Hunt:** Conceptualization (supporting); investigation (equal). **Ashleigh Courage:** Conceptualization (supporting); investigation (equal). **Stephanie Swanney:** Conceptualization (supporting); investigation (equal). **Stuart R. Dennis:** Writing – review and editing (supporting). **Dörthe Becker:** Writing – review and editing (supporting). **Stuart K. J. R. Auld:** Writing – review and editing (supporting). **Andrew P. Beckerman:** Conceptualization (lead); formal analysis (equal); investigation (equal); methodology (lead); supervision (lead); visualization (supporting); writing – review and editing (equal).

## FUNDING INFORMATION

The PhD student who carried out this research was funded by the Natural Environment Research Council through the IAPETUS doctoral training programme (S154).

## CONFLICT OF INTEREST STATEMENT

The authors have no conflict of interest to declare.

## Data Availability

The data that support the findings of this study are openly available in Dryad at https://doi.org/10.5061/dryad.zkh1893fx.
